# Behavioral Modulation and Molecular Definition of Wide-Field Vertical Cells in the Mouse Superior Colliculus

**DOI:** 10.1523/JNEUROSCI.1816-24.2025

**Published:** 2025-03-03

**Authors:** Xena J. Relota, Alexander Ford, Elise L. Savier

**Affiliations:** ^1^Molecular and Integrative Physiology Department, University of Michigan, Ann Arbor, Michigan 48109; ^2^Neuroscience Graduate Program, University of Michigan, Ann Arbor, Michigan 48109; ^3^Ophthalmology and Visual Science Department, University of Michigan, Ann Arbor, Michigan 48109

**Keywords:** cell types, locomotion, superior colliculus, vision

## Abstract

Visual information can have different meanings across species, and the same visual stimulus can drive appetitive or aversive behavior. The superior colliculus (SC), a visual center located in the midbrain has been involved in driving such behaviors. Within this structure, the wide-field vertical cells (WFV) are a conserved morphological cell type that is present in species ranging from reptiles to cats (Basso et al., 2021). Here, we report our investigation of the connectivity of the WFV, their visual responses, and how these responses are modulated by locomotion in male and female laboratory mice. We also address the molecular definition of these cells and attempt to reconcile recent findings acquired by RNA sequencing of single cells in the SC with the Ntsr1-Cre GN209 transgenic mouse line which was previously used to investigate WFV. We use viral strategies to reveal WFV inputs and outputs and confirm their unique response properties using in vivo two-photon imaging. Among the stimuli tested, WFV prefer looming stimuli, a small moving spot, and upward-moving visual stimuli. We find that only visual responses driven by a looming stimulus show a significant modulation by locomotion. We identify several inputs to the WFV as potential candidates for this modulation. These results suggest that WFV integrate information across multiple brain regions and are subject to behavioral modulation. Taken together, our results pave the way to elucidate the role of these neurons in visual behavior and allow us to interrogate the definition of cell types in the light of new molecular definitions.

## Significance Statement

Understanding how neuronal response preferences emerge remains a fundamental goal in neuroscience. Our ability to target neuron subpopulations and their embedding in circuits has greatly evolved over the last decades with the development of new tools including transgenic mouse lines and RNA sequencing methods. Here, we focus on wide-field vertical cells (WFV) which are found in the superior colliculus, a visual center in the midbrain that is highly conserved across species. Our findings challenge earlier definitions of this cell type and reconcile them with more modern approaches. Due to their conservation and connectivity, WFV present a model of choice to investigate how neurons gain their response specificity and relationships between structure, function, implication in behavior, and molecular profiles.

## Introduction

Individual survival relies on the identification of relevant cues followed by the generation of appropriate behavioral responses. This requires the detection of salient sensory information, such as object motion, which can indicate the presence of a prey or a predator. The superior colliculus (SC), a multisensory hub located in the midbrain, has been implicated in the detection of salient sensory information and generation of stimulus-driven behavioral responses. The SC is a prominent target of the retina whose superficial layers (sSC) are mostly visual (Basso and May, 2017; Cang et al., 2018). The organization of the SC is conserved across species, and the same morphologically defined cell types can be found across taxa (Luksch et al., 1998; May, 2006; Basso et al., 2021). While the role of the SC and its brain-wide connectivity is well characterized (Benavidez et al., 2021; Li et al., 2023), less is known about underlying cell types, their respective response properties, and behavioral modulation.

Earlier studies using Golgi staining have focused on the morphological and projection-based definition of neurons in the SC (Langer and Lund, 1974), while the later development of electrophysiology approaches provided functional definitions (Dräger and Hubel, 1975; Inayat et al., 2015; Franceschi and Solomon, 2018). Pioneer work from Gale and Murphy in the mouse suggests that four cell types can be found in the mouse SC: horizontal cells, stellate cells, narrow-field vertical cells, and wide-field vertical cells (WFV; Gale and Murphy, 2014, 2018). This work associated transgenic mouse lines with collicular cell types, among which the *Ntsr1*-Cre GN209 (Ntsr1-Tg) was associated with the WFV, and provided the foundation for multiple investigations (Shang et al., 2018; Hoy et al., 2019; Li and Meister, 2023). However, the recent generation of molecular-based cell atlases has uncovered a greater diversity of neuron types than previously characterized in the SC, with up to 28 molecularly defined cell types (Byun et al., 2016; Tsai et al., 2022; Liu et al., 2023). Here, we will revisit the definition of WFV, attempting to reconcile projection patterns, physiology, and molecular characterization.

WFV are conserved morphologically across species with somas located in the stratum opticum (SO), while their dendrites extend to the dorsal surface of the stratum griseum superficiale (SGS). WFV receive direct inputs from the retina, preferentially from αRGC and nondirection selective retinal ganglion cells (Reinhard et al., 2019; Tsai et al., 2022) and inputs from the primary visual cortex (V1; Masterson et al., 2019; Jiang et al., 2023). From a functional definition, WFV have large receptive fields, and previous studies in mice showed that they prefer small, slowly moving objects (Gale and Murphy, 2016). These visual responses are relayed to the lateral posterior nucleus of the thalamus or pulvinar, which is the sole target of the WFV (Gale and Murphy, 2014; Zhou et al., 2017).

Since WFV respond preferentially to motion, they have been suggested to contribute to the distinction between self-generated motion created by locomotion and externally generated motion created by the movement of objects (Roth et al., 2016; Beltramo and Scanziani, 2019; Brenner et al., 2023). To date, it remains unclear how locomotion modulates WFV visual responses, how WFV responses vary across the population, and how WFV labeled in the Ntsr1-Tg (Ntsr1+ SC neurons) correlate with newly identified molecular markers. The molecularly defined clusters that presumably correspond to the WFV do not express *Ntsr1* (neurotensin receptor 1), and expression atlases confirm that *Ntsr1* is not expressed in the SC (Liu et al., 2023). Here, we investigate WFV responses using in vivo two-photon calcium imaging, testing the hypothesis that the WFV labeled in the Ntsr1-Tg mouse line can be reconciled with newly identified molecular markers. We proceed with the characterization of WFV inputs and outputs, characterize Ntsr1+ SC neurons’ visual response properties and modulation by locomotion, and address their molecular identity. Our results show that the Ntsr1+ SC neuron population has an unexpected functional response diversity and stimulus-dependent modulation by locomotion and can be targeted using alternative molecular markers.

## Materials and Methods

### Animals

Results are reported for a total of 14 *Ntsr1*-GN209-Cre mice (Ntsr1-Tg, seven females and seven males, RRID:MGI:4367044), generously donated by Dr. M. Bickford and initially provided by Dr. G. Murphy, 8 Ntsr1-Tg crossed with a tdTomato reporter line (Ntsr1-Tg × tdTom, Ai14, RRID:ISMR_JAX:007914, or Ai9, RRID:IMSR_JAX:007909; four males and four females), and 3 wild types (C57BL/6j, RRID:IMSR_JAX:000664) aged 3–9 months. Ten animals were used for imaging and anterograde tracing (five females and five males) and four Ntsr1-Tg were used for retrograde tracing (two males and two females). Ntsr1-Tg × tdTom were used for anatomical tracings and histology. This does not include animals used for the optimization of these procedures or animals excluded due to either poor surgical quality which prevented imaging or lack of reporter expression. Animals were bred and raised in our animal facility, either at the University of Virginia or at the University of Michigan, and kept on a 12 h light/dark cycle, with 2–5 animals housed per cage prior to and after the surgery. All experimental procedures were approved by the University of Virginia and the University of Michigan Institutional Animal Care and Use Committee. No statistical methods were used to predetermine sample size, but our samples are in accordance with other similar studies. Due to our sample size, experiments were conducted in male and female mice and results were pooled together.

### Stereotaxic injection

Mice were anesthetized with isoflurane (5% induction; 2% maintenance in oxygen at 0.5 L/min, VetFlo, Kent Scientific) and prepared for surgery. Heads were shaved, a preventive analgesic (carprofen 5 mg/kg) was administered, and eye ointment was applied. Animals were then transferred and mounted on a stereotaxic frame (model 1900, Kopf Instruments, or model 68802, RWD Life Science), and the surgical site was disinfected using alternating rounds of ethanol and betadine solution. Upon confirmation of reflexes loss, an incision was made above the lambda point, and the periosteum was removed. Temperature was maintained using a feedback temperature controller and a heating pad (model 55, Harvard Apparatus, or DC, FHC). A small craniotomy was performed using a microdrill (model XL-230, Osada, or K.1070, Foredom) above the SC (0.5 mm anterior from the lambdoid suture and 0.5 mm lateral from the sagittal suture). Viral vectors were injected at 1.2 mm from the brain surface using a nanoliter injector (Nanoject II, Drummond Scientific) using a pulled glass capillary. To achieve the final target volume, 2.3 nl pulses were delivered every 10–20 s [50 nl for adeno-associated virus (AAV), 100 nl for pseudotyped rabies]. The viral titer was >10^12^ GC/ml for AAV1.Flx.Split.TVA.B19G (catalog #52473, Addgene, RRID:Addgene_52473-AAV1) and >10^8^ GC/ml for pseudotyped rabies virus (EnvA-coated G-deleted rabies-mCherry, Salk Institute, catalog #32636, Addgene, RRID:Addgene_32636). For anterograde tracings, 50 nl of AAV9.CAG.Flx.tdTomato was injected (catalog #28306, Addgene, RRID:Addgene_28306-AAV9). After injection, craniotomy was closed using bone wax, and the skin was sutured. Incubation time was 1 week for AAVs with CAG promoters, 3 weeks for other AAVs, and 7 d for pseudotyped rabies.

### Two-photon calcium imaging

The details of two-photon imaging procedures, including viral injections, surgery, imaging, visual stimulation, and data analysis, were provided in our previous publication (Savier et al., 2019). These procedures are only briefly described here, together with key parameters and novel technical details relevant to the current study. To image Ntsr1+ neurons in the SC, a 2.5 mm craniotomy was performed over the lambda point 2–3 weeks before imaging. During this procedure, AAV9.Syn.Flex.GCaMP6s viral vector (catalog #100845, Addgene, RRID: Addgene_#100845-AAV9) was injected into the SC at 500 and 250 µm below the surface. The craniotomy was closed using a glass window, and a small titanium plate was fixed to the skull. Five days after the surgery, mice were habituated to the imaging setup, head fixation, and running on the cylindrical treadmill. Functional data were acquired 2–4 weeks after surgery using a two-photon laser scanning microscope (Ultima Investigator or Ultima 2Pplus, Bruker Nano Surface Division) coupled to a Ti:sapphire laser (Chameleon Discovery with or without TPC, Coherent, or Vision II, Coherent) at 920 nm using a 16×, 0.8 NA Nikon objective. The microscope was controlled using PrairieView software (Version 5.4 or 5.8), images were acquired using resonant scanning at 30 Hz using 2× optical zoom at 512 × 512-pixel resolution, and four-frame averages were used for analysis.

### Visual stimuli

Similar to our previous studies (Savier et al., 2019), visual stimuli were generated using the MATLAB Psychophysics toolbox (Brainard, 1997; RRID:SCR_002881). An LCD monitor was placed 25 cm away from the eye contralateral to the imaging site and aligned with the visual receptive field of the imaged location, corresponding to the dorsal–caudal portion of the visual field. Drifting grating parameters were similar to previous studies (100% contrast, 0.08 cpd, 2 Hz, 40° diameter, 12 directions in 30° increments, presented for 1 s, with an interstimulus interval consisting of a gray screen for 3 s, pseudorandomized conditions). Zero degree represented forward motion from the animal's perspective (temporal to nasal). For the flashing spot, a 2° black square was presented at a pseudorandom location on a grid of 20 × 20° for a duration of 1 s, with an interstimulus interval of 3 s, with a minimum of five repeats. The moving spot was also a 2° black square moving across the monitor at a speed of 30°/s across for 40° of the field of view moving along the cardinal directions and starting at random edge locations. For moving dots, 25 dots of a 2° size with random spacing and a view size of 40° diameter were displayed and moved at a speed of 30°/s in 12 directions and presented for 1 s and with a blank consisting of a static image of the generated random dots for 3 s. Looming stimulus consisted of a black expanding disk on a gray background from 0 to 60° with variable expansion speed (from 10 to 40°/s, 10° increments). Synchronization and condition identification were performed by recording voltage commands generated by the Psychophysics toolbox using the PrairieView software. Locomotion was measured as the rotation of the cylindrical treadmill using a rotary encoder generating transistor–transistor logic pulses (100 pulses/revolution).

### Analysis of two-photon calcium imaging data

We followed our published procedures to analyze the imaging data. Briefly, ROIs were manually drawn and Δ*F*/*F*_0_ = (*F* − *F*_0_)/*F*_0_ was calculated, where *F*_0_ was the average fluorescence signal over six frames before stimulus onset and *F* was the average fluorescence signal over eight frames, with one frame after stimulus onset, and the average response across multiple trials was used. The mean value of Δ*F*/*F*_0_ for each stimulus condition was then used for subsequent data analysis for all the responsive neurons. A neuron was considered responsive if its mean Δ*F*/*F*_0_ at the preferred condition was >0.2. To determine stimulus specificity, a Pearson’s correlation coefficient was calculated between the responses for different types of stimuli: $\rho X,Y=\frac{\mathrm{cov}(X,Y)}{\sigma X\sigma Y}$, where cov is the covariance, *σ_X_* is the standard deviation of *X* and *σ_Y_* is the standard deviation of *Y*.

To determine if the animal was running or stationary, trials were sorted as a function of the animal speed prior to and during stimulus presentation using 1 cm/s as a threshold.

The effect of locomotion was quantified using a locomotion modulation index (LMI):

$$\mathrm{LMI}=\frac{R_{\mathrm{running}}-R_{\mathrm{stationary}}}{R_{\mathrm{running}}+R_{\mathrm{stationary}}},$$

where *R*_running_ is the average response during running condition and *R*_stationary_ is the response during stationary condition. As previously reported, trial-to-trial variability was analyzed using the Fano factor $F=\frac{\sigma^{2}}{\mu }$ (i.e., the variance of the response magnitudes over all trials divided by the mean). To determine the response selectivity of neurons to the direction of motion, we used the global direction selectivity index (gDSI), which is the vector sum of Δ*F*/*F*_0_ responses normalized by their scalar sum:

$$\mathrm{gDSI}=\frac{\sum R_{\theta }e^{i\theta }}{\sum R_{\theta }},$$

where *R* is the response magnitude in Δ*F*/*F*_0_ at direction *θ* of the stimulus (Gale and Murphy, 2014).

### Immunostaining

Mice were deeply anesthetized with a lethal dose of pentobarbital and then perfused transcardially with phosphate buffer saline (PBS) followed by 10% neutral buffered formalin (NBF). Brains were harvested and postfixed overnight in NBF. Fifty micrometer brain sections were obtained using a vibratome (VT1000, Leica). Sections were incubated for 1 h in blocking buffer (bovine serum albumin 1%, donkey serum 10%, Triton X-100 0.5% in PBS), before being incubated in primary antibody solution (chicken anti-synaptophysin 1 at 1:1,000, Synaptic Systems catalog #101 006, RRID:AB_2622239; guinea pig anti-VGluT2 at 1:750, Synaptic Systems catalog #135 418, RRID:AB_2864786) overnight at 4°C or 2 h at room temperature. Following primary incubation, sections were washed in PBS for 5 min three times and then incubated in secondary antibody solution (donkey anti-chicken Alexa Fluor 647 at 1:1,000, Thermo Fisher Scientific catalog #A78952, RRID:AB_2921074; donkey anti-guinea pig FITC at 1:1,000, Millipore catalog #AP193F, RRID:AB_92670) for 1 h 30 min. All antibodies were diluted in incubation buffer (BSA 1%, donkey serum 5%, Triton X-100 0.5% in PBS). After secondary incubation, sections were washed in PBS for 5 min twice and then mounted on microscope slides (Superfrost Plus, Fisherbrand) with mounting medium containing DAPI (Fluoromount-G, Invitrogen). Slides were imaged on a Nikon A1R confocal microscope.

### In situ hybridization

Mice were killed with a lethal dose of pentobarbital, and after decapitation, brains were immediately harvested and flash frozen on dry ice. Frozen specimens were stored up to 3 d at −70°C before sectioning. Sixteen micrometer coronal cryosections of the SC were obtained using a cryostat (CM3050s, Leica) and directly mounted onto microscope slides (Superfrost Plus, Fisherbrand). Sections were postfixed in 10% NBF for 1 h, followed by dehydration in increasing concentration of ethanol (50, 70, 100%). RNAscope Multiplex V2 (catalog #323100, ACDBio) was then performed on sections following manufacturer instructions. Briefly, sections were treated with 10% H_2_O_2_ to quench endogenous peroxidase activity and digested with protease IV (catalog #322340, ACDBio). Endogenous mRNA was then hybridized with target probes* Cbln2* (428551-C1), tdTomato (317041-C2), and *Necab1* (428541-C3); *Cbln2* (428551-C1), *Npnt* (316771-C2), and *Necab1* (428541-C3); or tdTomato (317041-C2) and* Npnt* (316771-C1) at 40°C for 2 h and then stored in 5× saline–sodium citrate (SSC) overnight. The next day, probes were amplified with Amps 1–3, and the signal was developed using HRP C1/C2/C3 and its blocker to bind fluorophores (TSA Vivid 520/570/650 diluted 1:2,000 and 1:1,500 for 570) to their respective channels. Sections were washed for 5 min after each step using an in-house wash buffer (0.1× SSC, 0.03% lithium dodecyl sulfate). After the assay, sections were mounted using a mounting medium containing DAPI (Fluoromount-G, Invitrogen) and imaged on a Nikon A1R confocal microscope.

### Anatomical tracing histology

For anatomical tracing, samples were initially prepared similarly to the immunostaining procedure. After sectioning, slices were directly mounted on microscope slides (Superfrost Plus, Fisherbrand) with a mounting medium containing DAPI (Fluoromount-G, Invitrogen, or Vectashield, Vector Laboratories). Slides were imaged using a confocal microscope (Nikon A1R confocal microscope or Zeiss LSM 800).

### Histology quantification

#### In situ hybridization

Fluorescent in situ hybridization staining photomicrographs of the SC were acquired at 40× as *Z*-stacks using a Nikon A1R confocal. Both hemispheres, medial and lateral portions, of the SC were acquired and analyzed independently. Areas where the slices were damaged were excluded. All quantifications were performed in Fiji (Schindelin et al., 2012) as follows. After projecting the *Z*-stacks, regions of interest (ROIs) were drawn around cells with high expression (>10 puncta) using the ROI manager plugin (Fiji), and the ROI mask was saved. The ROI mask was then applied on the *Z*-stack, and the cell counter tool (Fiji) was used to classify each ROI based on puncta density in each channel. A threshold of four small or two large puncta was used to classify ROIs as positive or negative for each respective channel and probe. To account for channel bleed-through, low-intensity puncta that perfectly overlapped across channels were excluded. ROIs from each experiment were classified as expressing one of the three probes, a combination of two, or all three probes. Counts for each of these seven classification groups were compiled for each experiment, animal, and subregion. The average of all subregions was taken for each classification, and then these averages were averaged across all animals for the experiment. Venn diagrams of these quantities were made using Eulerr (Larsson and Gustafsson, 2018).

#### Immunostaining

Photomicrographs of the immunostaining were acquired as *Z*-stacks at 60× using a Nikon A1R confocal, and these *Z*-stacks were projected using maximum intensity. The pulvinar was used as a positive control due to the known presence of excitatory synapses (Zhou et al., 2017). Putative synapses were identified using the tdTomato channel by selecting ROIs with bouton-like shapes of ∼1 µm size. To determine if ROIs were synapses and if they were excitatory, the immunofluorescent labeling of VGluT2 and synaptophysin channels was examined. If labeling was sufficiently intense and resembled the shape of the bouton, the ROI was classified as positive for the respective label. ROIs were classified as expressing either VGluT2 or synaptophysin, both (colocalized), or neither (unstained). The number of ROIs in each of the four groups was counted for both the pulvinar and parabigeminal nucleus (PBG) of each animal, and means were calculated across all four animals.

#### Laminar distribution

For the depth profile, analysis was performed using custom MATLAB code. Confocal images were oriented anatomically and between 100 and 150 lines spaced by 20 µm were drawn from the dorsal tissue surface, perpendicular to this surface and to a depth of 1 mm across the entirety of the SC. Raw pixel gray-value intensity was extracted per fluorophore channel and fluorescence intensity values were averaged across columns. The minimum-value background was then subtracted and normalized by the DAPI channel to account for variable densities in cells. Results were smoothed using a moving window average of approximately 20 µm (∼1 cell width) and plotted as a function of the distance from the tissue dorsal surface.

### Experimental design and statistics

Significance was calculated using two-sided statistical tests including Wilcoxon signed rank tests and Pearson’s correlation coefficient as stated. All analyses and graph plotting were performed in MATLAB (MathWorks; RRID:SCR_001622) or R (R Foundation for Statistical Computing; RRID:SCR_001905) using custom scripts, which are all available upon request. No statistical methods were used to predetermine sample sizes, but our sample sizes are similar to those reported in the field. We did not randomly assign animals to groups because it is not applicable to the experimental design of this study.

## Results

### The majority of Ntsr1+ SC neurons are visually responsive and prefer small moving objects

**Figure 1.**
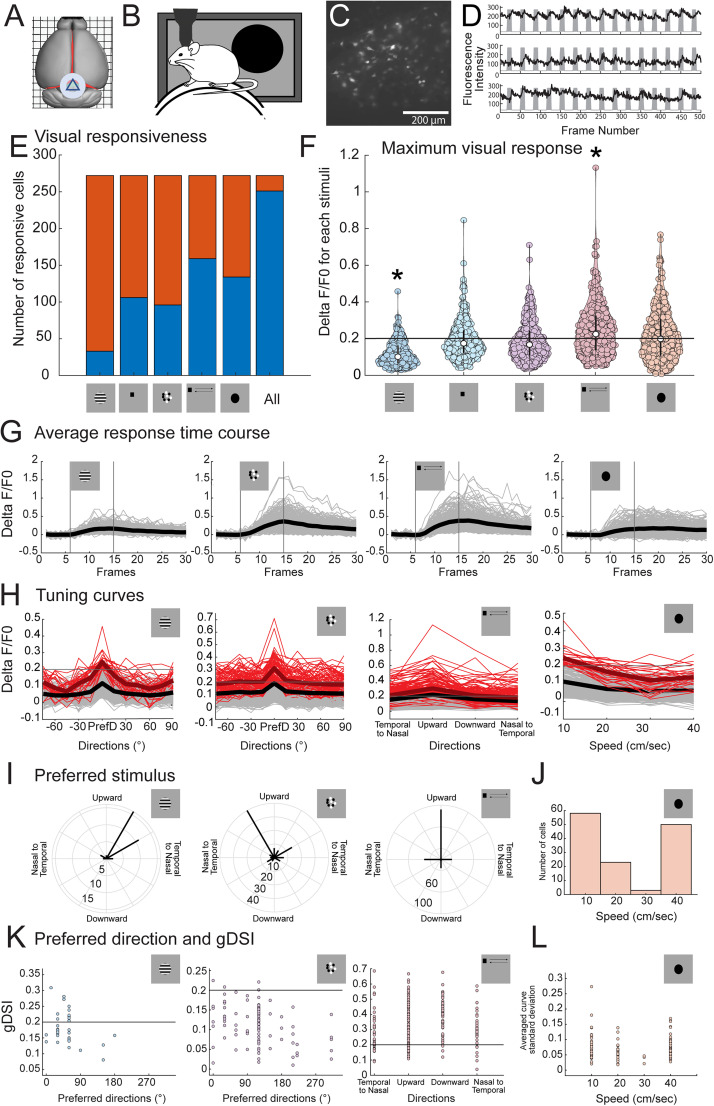
Ntsr1+ SC neurons visual response properties. ***A***, Schematic depicting the location of the cranial window for in vivo imaging of the SC. ***B***, Schematic of the imaging setup. Stimulus was displayed on a movable LCD screen, aligned with the neurons’ receptive fields. ***C***, Representative field of view at 300–400 µm below the SC surface, where Ntsr1+ SC neuron somas are located. ***D***, Representative traces of three different Ntsr1+ SC neurons. Shaded areas represent time periods during which the visual stimulus was presented, here a moving spot. See Materials and Methods for details. ***E***, Distribution of visually responsive neurons depending on the stimulus type (Five mice, 272 neurons). Unresponsive neurons are in orange, and responsive neurons are in blue. ***F***, Distribution of visual responses of Ntsr1+ SC neurons for different types of stimuli at the condition that elicited the highest average response. Drifting gratings and moving spot significantly differ from other stimuli, 10 comparisons, Wilcoxon signed rank test, *p* < 0.005, indicated with a star. ***G***, Average response time courses per neuron during the presentation of a visual stimulus. The vertical lines denote the beginning and end of the stimulus presentation. The gray lines represent individual neuron average responses to the presentation of their preferred condition for each type of stimulus, and the black line shows average response across neurons. ***H***, Tuning curve for each neuron. The gray lines represent individual tuning curves for all neurons, and the black line is the average tuning curve across neurons. Red and dark red are tuning curves for neurons that are considered visually responsive. For drifting gratings and moving dots, curves were aligned to the preferred direction, while for the moving spot and looming, curves are plotted as a function of condition. ***I***, Distribution of Ntsr1+ SC neurons preferred direction for drifting gratings (left panel), moving dots (central panel), and moving spot (right panel). ***J***, Distribution of Ntsr1+ SC neurons preferred expanding speed for a looming stimulus. ***K***, Visually responsive neurons preferred direction as a function of global direction selectivity index in response to drifting gratings, moving dots, and moving spot. The horizontal line depicts a 0.2 cutoff, usually employed to determine highly selective neurons. ***L***, Neuron preferred looming speed as a function standard deviation for averaged tuning curves for visually responsive neurons.

To study Ntsr1+ SC neurons’ visual responses, we performed in vivo two-photon calcium imaging in Ntsr1-Tg mice. Briefly, a cranial window was implanted above the caudal–medial pole of the SC, which corresponds to the upper, caudal part of the visual field (Fig. 1*A*), and a Cre-dependent genetically encoded calcium indicator (GCaMP6s) was expressed using a viral vector. Mice were habituated and head-fixed atop a cylindrical treadmill, and a screen was aligned to the visual field corresponding to the imaged location (Fig. 1*B*). The screen allowed the presentation of different visual stimulus types including sinusoidal drifting gratings, moving dots, a single moving spot, a single flashing spot, and expanding disk of variable parameters (see Materials and Methods for details). Example field of view and responses are shown in Figure 1, *C* and *D*, respectively.

Overall, Ntsr1+ SC neurons were responsive to at least one type of visual stimuli (251/272, 92%, Fig. 1*E*). Two criteria for responsivity were initially tested, either a threshold of 0.2 Δ*F*/*F*_0_ for the average response across trials or a bootstrap analysis. Both approaches yielded a similar percentage of responsive cells (<3% difference, data not shown). As such, a 0.2 Δ*F*/*F*_0_ cutoff was chosen for further analysis in determining if a neuron is responsive to a particular stimulus condition. Here, we present the results for 272 Ntsr1+ SC neurons obtained from five mice, from a single imaging session, in which 34, 37, 64, 53, and 84 Ntsr1+ neurons were recorded, respectively, per animal. We proceeded with the identification of which stimulus type elicited the highest responses (Fig. 1*F*), and within each stimulus type, we identified which parameters were preferred (Fig. 1*I*,*J*). The preferred type of visual stimuli was the single moving spot, for which the preferred condition elicited the highest Δ*F*/*F*_0_ across all conditions and stimuli. Fifty-eight percent of neurons (159/272) were responsive to the presentation of a single moving spot, while 49% (134/272) were responsive to looming. When comparing visual responses evoked by the different stimuli, only the drifting grating and moving spot elicited significantly lower and higher responses, respectively (10 comparisons, Wilcoxon signed rank test, *p* < 0.005). The average response per neuron for each stimulus condition as a function of time confirms that the presentation of a visual stimulus indeed elicited an increase in fluorescence in Ntsr1+ SC neurons (Fig. 1*G*), confirming their visual responsivity. Tuning curves (Fig. 1*H*) show that Ntsr1+ SC neurons show some degree of orientation selectivity in response to the presentation of a drifting grating and are mostly responsive to their preferred direction for the moving dots and the moving spot. These results show that Ntsr1+ SC neurons show heterogeneous visual response preferences and prefer on average a small discrete moving object. These results are in agreement with previous studies (Gale and Murphy, 2014, 2016). We next addressed if some parameters within these stimulus types, such as direction or speed, might affect these response preferences.

### Ntsr1+ SC neurons show similar response preferences within types of visual stimuli

When presented with drifting gratings, the majority of Ntrs1+ SC neurons were weakly responsive, and in three out of five mice, responses were below our responsivity criteria. In the two animals where responses were elicited, only 26% of neurons were responsive to drifting gratings (33/126 neurons), and 81% showed a strong preference for upward direction of grating (27/33, either 30 or 60°; Fig. 1*I*, left panel). When examining all animals, the average Δ*F*/*F*_0_ for drifting gratings was 0.11. Overall, Ntsr1+ SC neurons are weakly responsive to drifting gratings and show a bias for upward direction. These weaker responses to large drifting gratings could be linked to stimulus size (bars vs dots), similar to what was previously reported (Gale and Murphy, 2016).

Responsivity was slightly stronger when using moving dots, for which responsive cells could be found in all imaged animals (96/272 neurons), with an average Δ*F*/*F*_0_ of 0.19 (Fig. 1*F*,*G*). Note that all responses to the different stimuli were recorded within the same session, and stimulus presentation size was the same for drifting gratings and moving spots, thus preventing misalignment between the imaged area and the visual field across sessions. Interestingly, Ntsr1+ SC neurons also showed preference for upward direction with moving dots, with 70% of the population preferring between 150 and 30°, with a bias for 120° (40/96 neurons; Fig. 1*I*, center panel).

When using a single moving spot, which was presented with varying starting locations within the visual field, a bias toward the upward direction could also be observed (92/152 neurons; Fig. 1*I*, right panel). While response intensity varied depending on the type of stimulus used, Ntsr1+ SC neurons located in the medial caudal pole of the SC, which corresponds to the upper caudal visual field, preferred the upward direction of motion.

Due to its ethological relevance, and previous studies implicating Ntsr1+ SC neurons in escape behavior, we tested the specificity of Ntsr1+ SC neurons responses to the speed of expansion of a disk, which mimics an approaching object. A strong preference was observed for either the slowest and highest speed of expansion presented (Fig. 1*J*). We next addressed if any relationship could be found between the selectivity of response and stimulus preference. To this end, we calculated the global direction selectivity index (see Materials and Methods) for responses to the drifting gratings, moving dots, and moving spots. No clear relationship could be established between stimulus preference and response selectivity (Fig. 1*K*). For the looming stimulus, we used the standard deviation of the average curve as an indication of selectivity since a strong preference for a particular speed would result in a higher standard deviation. Similarly to visual stimuli with motion direction, no clear relationship could be established between selectivity and response preference (Fig. 1*L*). While not all Ntsr1+ SC neurons prefer the same type of stimulus, they tend to prefer the same parameter within a type such as speed or direction. We next addressed if neurons that are highly responsive to some stimuli are highly responsive to all stimulus types.

### Ntsr1+ SC neurons display different response intensities between stimulus types

**Figure 2.**
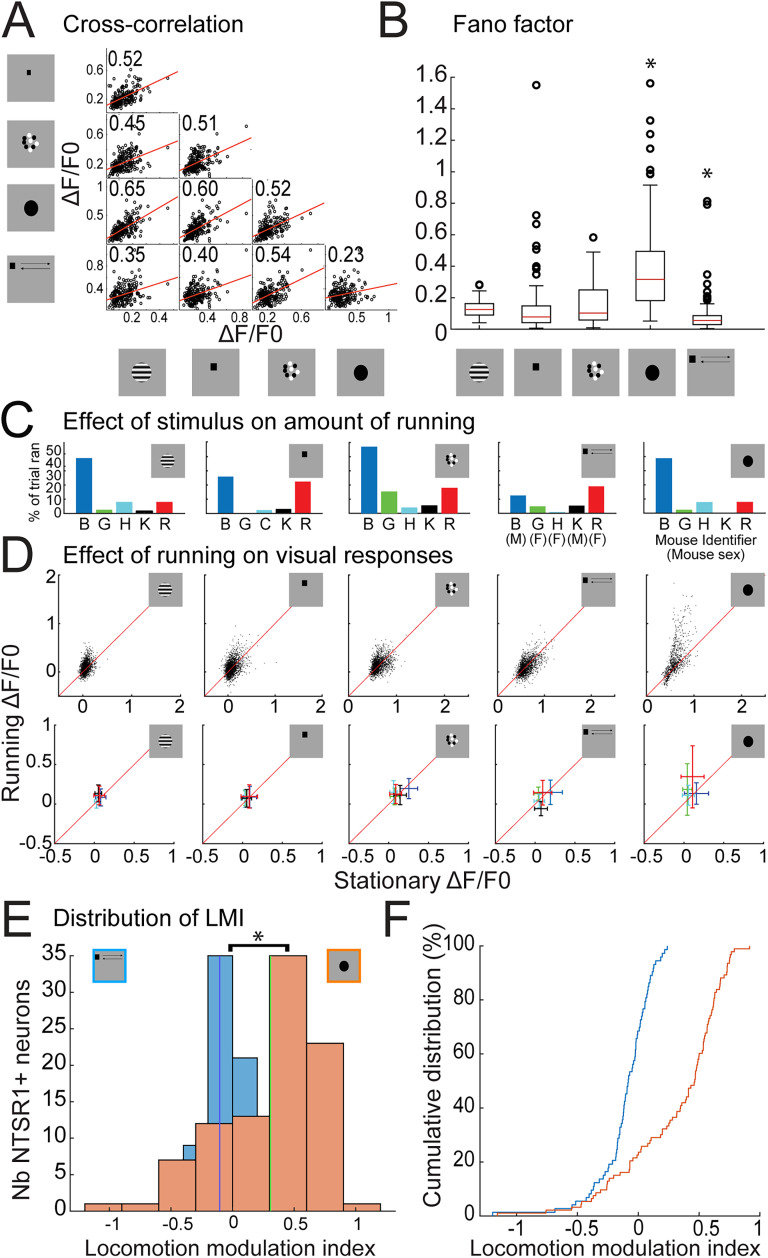
Ntsr1+ SC neurons’ visual responses are variable and modulated by locomotion. ***A***, Correlation between the responses of the same cell during the same imaging session for different types of stimuli. Analysis was performed for the parameters that elicited the highest response within a stimulus type. ***B***, Response variability for each type of visual stimulus. Drifting gratings, flashing spot, and moving dots did not show significant differences in variability between each other, while looming and moving spot were significantly different (Wilcoxon signed rank test, 10 comparisons, *p* < 0.005, indicated with a star). ***C***, Percentage of trials individual animals spent running as a function of stimulus type. Animal identifier and sex are also specified in the two rightmost panels. ***D***, Visual responses for all stimuli and all parameters during stationary and running conditions. The top row shows individual responses per neuron, per condition, while the bottom row shows average responses and variability for each animal. Note that only four out of five animals ran for the drifting grating and looming stimulus sets. ***E***, Distribution of the locomotion modulation index in response to the presentation of a moving spot and a looming stimulus. Distributions are significantly different (Wilcoxon signed rank test, *p* < 0.05, indicated with a star). ***F***, Cumulative distribution of the LMI. Five mice, 272 neurons.

Upon the observation that some cells are highly responsive to certain stimulus conditions, we asked if the same cells were highly responsive across all stimuli or if some cells showed preference for some over the other. To this end, we calculated the correlation coefficient at the preferred condition between stimulus types (Fig. 2*A*). If a Ntsr1+ SC neuron is highly responsive for all types of stimuli, the correlation coefficient will be high, while if a neuron can be selective to a type of stimuli, the correlation will be low. Interestingly, we found the lowest correlation to be between responses for looming and the small moving spot (*r* = 0.23, *p* = 0.0007), suggesting that Ntsr1+ SC neurons that are highly responsive to looming are not necessarily highly responsive to a small moving spot. This observation could be explained by other factors contributing to Ntsr1+ SC neuron response properties, which could modulate responses to a specific stimulus type. To this end, we next evaluated the response variability as a function of stimulus.

### Ntsr1+ SC neurons show variability between stimuli which correlates with locomotion

For each Ntsr1+ SC neuron that was visually responsive, we evaluated the response variability for each stimulus type at the preferred condition. Interestingly, we found that the Fano factor, which is the variance squared divided by the mean, is the lowest for the small moving spot, while it is the highest for looming (median = 0.05 and 0.3171, respectively). The response variability was not significantly different between the responses evoked by the drifting gratings, flashing spot, and moving dots, while looming and moving spot displayed different distributions (10 comparisons, Wilcoxon signed rank test, *p* < 0.005, Fig. 2*B*). Other stimulus types had a median Fano factor of approximately 0.1. These results show that Ntsr1+ SC neurons’ responses are more variable when a looming stimulus is presented and more stable in response to a small moving spot.

Locomotion has been shown to modulate visual responses in multiple brain regions, including the SC, where it can differentially impact cell types (Savier et al., 2019; Schröder et al., 2020, Ito et al., 2017). Here, we tested the hypothesis that the increased response variability to looming stimuli is caused by locomotion. To this end, we paired responses to a specific stimulus condition between the running and the stationary condition for each stimulus type. The condition was classified as running if the animal was running during both the baseline and the stimulus presentation. Due to this criterion, only four animals spent enough time running across conditions for this analysis during the presentation of the looming and drifting grating stimuli. Overall, animals ran on average 11 ± 14% of the trials during the presentation of the moving spot, 18 ± 17% of the time during the presentation of moving dots, 8 ± 7% of the time during flashing spot, 10 ± 12% during drifting gratings, and 10 ± 10% of trials during looming. Mice showed a high variability in the amount of time spent running; however, they did not run more during the presentation of a specific type of stimulus (Fig. 2*C*). Surprisingly, we found significantly different effects on visual responses depending on the stimulus type (Fig. 2*D*). While visual responses were mildly modulated by locomotion when visual stimuli were presented (mean LMI for all stimulus = 0.0315), a significant increase was seen specifically when a looming stimulus was presented (57/71 conditions that had twofold increase in visual responses were in response to looming).

For further analysis, we chose to focus on the modulatory effect of running on the moving spot and the looming since these two stimulus types evoked the strongest visual responses in Ntsr1+ SC neurons and have been previously shown to evoke behavioral responses (De Franceschi et al., 2016). We calculated a locomotion modulation index to address the distribution of this modulation for visually responsive Ntsr1+ SC neurons. Since not all neurons and conditions occurred due to our responsive and running criteria, here we report results for 73 neurons for the moving spot and 93 neurons for the looming stimulus for five and four mice, respectively. A negative index indicates a reduction in fluorescence intensity when the animal is running during stimulus presentation, while a positive index indicates an increase. Overall, the majority of visually responsive Ntsr1+ SC neurons are significantly modulated by running during looming presentation, with 57/93 neurons displaying a twofold increase in their response, with a mean LMI of 0.3. It is worth noting that this effect is mostly driven by two out of the four animals as displayed by the animal average response (Fig. 2*D*). For the moving spot, none of the analyzed visually responsive neurons showed a twofold increase, and the mean LMI was −0.105, reflecting a decrease in visual responses. The LMI for the moving spot and looming differed significantly (Wilcoxon signed rank test, *p* < 0.05; Fig. 2*E*,*F*). Overall, Ntsr1+ SC neurons’ visual responses are mostly modulated by locomotion during the presentation of a looming stimulus. This type of stimulus has behavioral significance since it mimics an approaching object and triggers defensive behavior. We next addressed the specificity of expression of Ntsr1+ neurons beyond the SC.

### Ntsr1-Cre expression can be found in multiple brain regions

**Figure 3.**
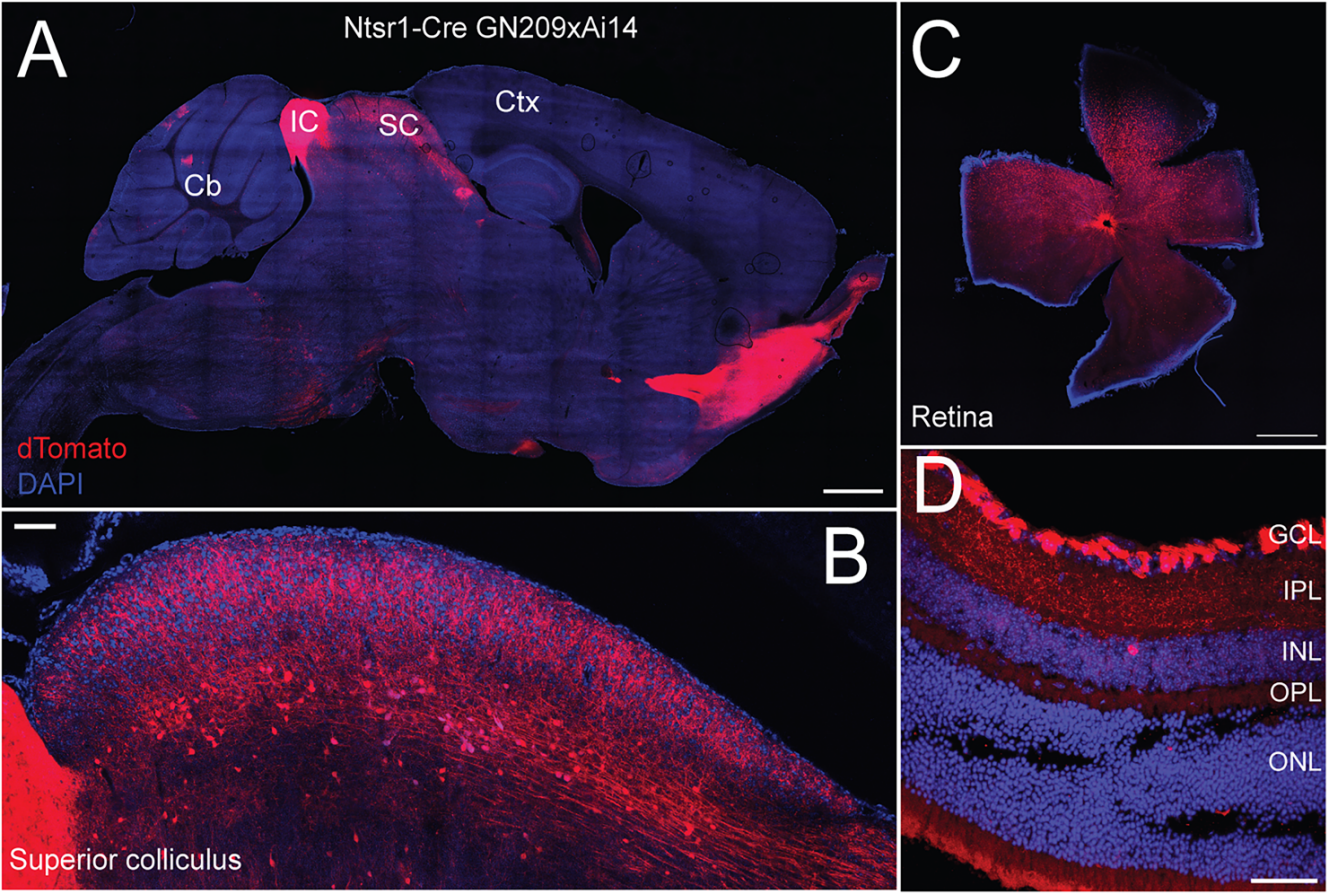
Brain-wide expression of tdTomato in the Ntsr1-TgxAi14 mouse line. ***A***, Sagittal section of an Ntsr1-Tg mouse crossed with the reporter strain Ai14, leading to Cre-dependent expression of tdTomato. Note the high expression in the inferior colliculus. Scale bar, 1,000 µm. ***B***, Sagittal section of the superior colliculus from the same cross as in ***A***. Scale bar, 100 µm. ***C***, Flat-mounted retina of an Ntsr1-TgxAI14. tdTomato reporter expression can be found throughout the retina. Scale bar, 500 µm. ***D***, Cryosection of a Ntsr1-TgxAi14 retina. Expression is mostly located in the ganglion cell layers. Scale bar, 50 µm. Eight mice. Cb, cerebellum; Ctx, cortex; GCL, ganglion cell layer; IC, inferior colliculus; INL, inner nuclear layer; IPL, inner plexiform layer; ONL, outer nuclear layer; OPL, outer plexiform layer; SC, superior colliculus.

To assess the specificity of Cre expression in the Ntsr1-Tg mouse line, we characterized the distribution of Ntsr1+ cells throughout the early visual system and the midbrain. We crossed the Ntsr1-Tg mouse line with a reporter line, the Ai14 or Ai9, in which tdTomato expression is Cre-dependent and examined expression in eight animals. This allowed the visualization of neurons which had, at some point during development, expressed the recombinase Cre. As previously described, we found tdTomato expression to be mostly restricted to the SO layer of the superficial SC, with some expression in the deeper layer of the SC (Fig. 3*A*,*B*). High expression of the reporter could also be found in the neighboring inferior colliculus, cerebellum, and anterior brain regions. We also examined the expression of the reporter in the retina since Ntsr1+ neurons have been previously reported to receive direct inputs from the retina (Tsai et al., 2022). Surprisingly, we found expression throughout the retina (Fig. 3*C*) and more specifically restricted to the retinal ganglion cell layers (Fig. 3*D*). Having characterized functional and anatomical properties of Ntsr1+ SC neurons, the next step was to reveal inputs to these neurons that could be implicated in the locomotion modulation observed.

### Ntsr1+ SC neurons receive inputs from multiple brain regions

**Figure 4.**
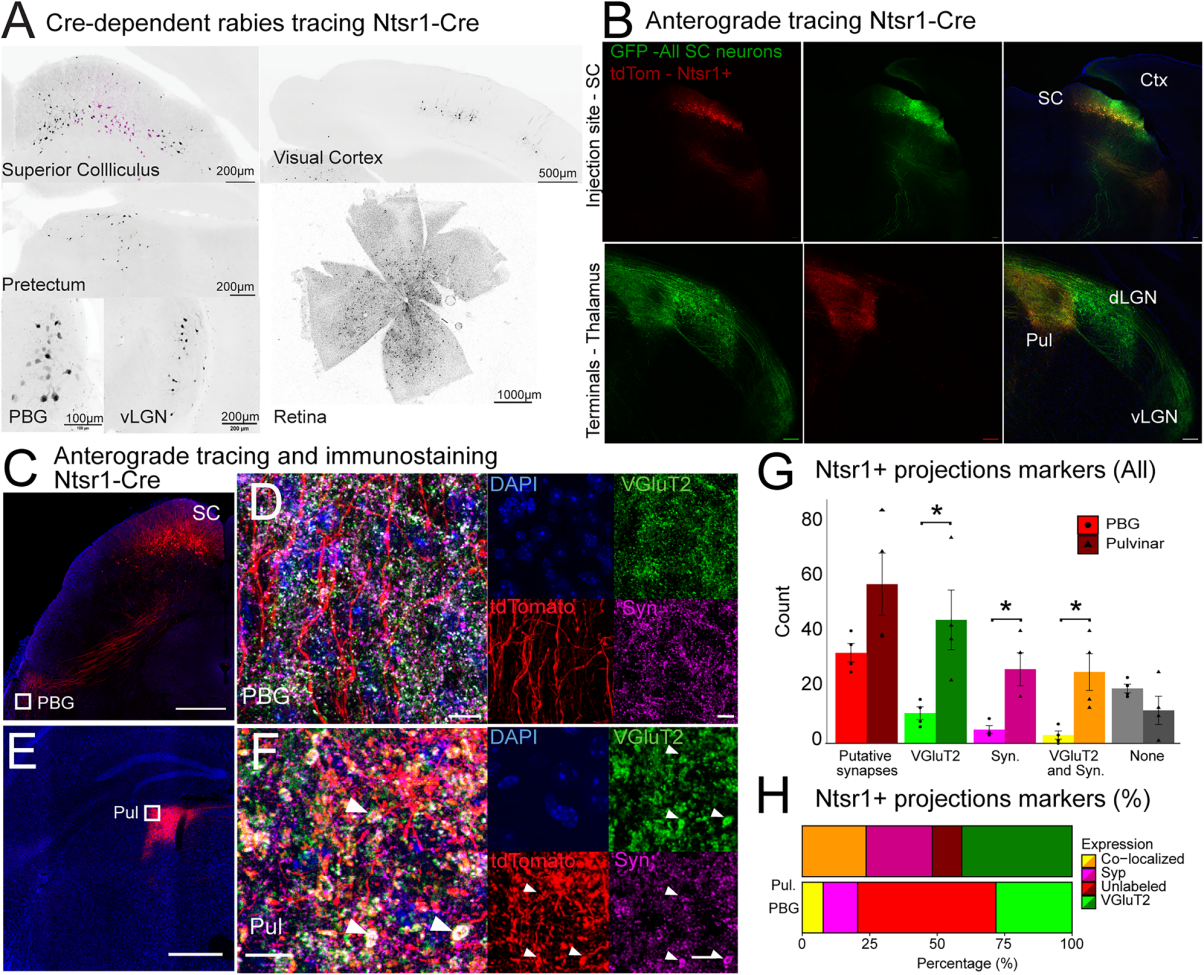
Ntsr1+ SC neurons input and output characterization. ***A***, Photomicrographs of Cre-dependent pseudotyped rabies tracing of Ntsr1+ neurons. Starter cells in the SC are highlighted in magenta. Cell bodies were identified in the visual cortex, retina, pretectum, parabigeminal nucleus (PBG), and ventral lateral geniculate nucleus (vLGN). Three mice. ***B***, Photomicrograph of the injection site and projection pattern of Ntsr1+ neurons in the midbrain after injection of an AAV carrying Cre-dependent tdTomato (Ntsr1+ neurons) together with an AAV carrying CAG-driven GFP (all SC neurons). The top row shows the injection site, and the bottom row shows projections in the thalamus. Scale bar, 100 µm, three mice. ***C***, Photomicrograph of the injection site and projection pattern of Ntsr1+ neurons in the midbrain after injection of an AAV carrying Cre-dependent tdTomato only. ***D***, Immunolabeling of the projections of neurons labeled in ***C*** for VGluT2 and synaptophysin. ***E***, ***F***, Same as ***C*** and ***D*** for the thalamus, showing the pulvinar and corresponding inputs from the Ntsr1+ SC neurons. Scale bars: ***C*** and ***D***, 500 µm; ***D*** and ***F***, 10 µm. ***G***, Absolute quantification of putative synapses originating from Ntsr1+ neurons in the SC and their immunolabeling in the pulvinar and PBG. Wilcoxon signed rank test, *p* < 0.05, indicated with a star. ***H***, Proportion of putative synapses labeled in the PBG and pulvinar. Four mice.

To further characterize the inputs that Ntsr1+ SC neurons receive, we used a G-deleted rabies viral strategy and injected Ntsr1-Tg mice first with an AAV (adeno-associated virus) expressing G-protein and a TVA receptor in a Cre-dependent manner. The TVA receptor (avian receptor) provides mammalian cells the capacity to be transfected by EnvA-coated rabies particles, which otherwise cannot transfect mammalian cells. The glycoprotein is necessary for the G-deleted rabies to propagate retrogradely across synapses. The AAV expressed in addition mCitrine to allows for the visualization of transfected cells. After 3 weeks, the EnvA-coated G-deleted rabies expressing mCherry was injected and allowed to express for 1 week (Fig. 4*A*). Animals were then perfused and processed for histology. Brain-wide analysis was performed in three animals and revealed the presynaptic inputs to the Ntsr1+ SC neurons. As previously described (Reinhard et al., 2019; Tsai et al., 2022), Ntsr1+ SC neurons receive inputs from the retina and visual cortices, both primary and higher visual areas (Fig. 4*A*, top right). In addition, we were able to identify inputs from additional brain regions that had not been reported in the mouse, such as the pretectum, the vLGN, and the parabigeminal nucleus (PBG; Fig. 4*A*). These results suggest that Ntsr1+ SC neurons receive inputs from a broader selection of areas than previously reported. In addition to previously identified inputs from the retina and cortical areas, Ntsr1+ SC neurons receive inputs from the PBG, vLGN, and pretectum, which could be implicated in their modulation by locomotion.

### Ntsr1+ SC neurons project to the pulvinar

Previous studies have reported that WFV provide the sole source of collicular inputs to the pulvinar (LP) in mice (Gale and Murphy, 2014). To confirm that Ntsr1+ SC neurons project exclusively to the pulvinar, we further analyzed the projection pattern of Ntsr1+ SC neurons from the SC (Fig. 4*B*). We performed injections in the SC of Ntsr1-Tg mice of an AAV carrying Cre-dependent tdTomato together with an AAV carrying non-Cre-dependent GFP, thus allowing the visualization of Ntsr1+ SC projections in red and all SC neurons in green throughout the brain. The injection site in the SC can be seen in the top row of Figure 4*B* while projections in the thalamus can be seen in the bottom row. While we confirmed previously known targets of the sSC in the thalamus such as vLGN and dLGN, for all SC neurons, Ntsr1+ SC neurons projections were restricted to the pulvinar (Fig. 4*B*, bottom row).

To investigate the presence of synapses where Ntsr1+ SC neuron projections could be observed, we injected the SC of Ntsr1-Tg animals with an AAV carrying Cre-dependent tdTomato and proceeded with immunostaining of synaptic markers. Since fibers can be seen passing in proximity to the PBG and seem to end in the pulvinar, we performed synaptophysin and VGluT2 immunostaining to confirm the presence of synapses in both regions (Fig. 4*D*,*F*) and confirm that Ntsr1+ SC neurons projections are glutamatergic. Summarized in Figure 4, panels C and E show an example animal, showing the injection site in the SC and the corresponding terminals of Ntsr1+ SC neurons in the pulvinar and PBG. Ntsr1+ SC neurons projected bilaterally to the pulvinar as previously reported by Naeem et al., (2022). An overlap between fibers and immunolabeled puncta could be found on the pulvinar, while a reduction in overlap was found in the PBG (twofold increase in overlap between synaptophysin, VGlut2, and tdTomato expressing Ntsr1+ terminals between pulvinar and PBG, three mice; Fig. 4*G*,*H*). We further confirmed that Ntsr1 projections to the pulvinar are glutamatergic (Fig. 4*D*,*F*; quantified in panels G and H). The amount of putative synapses was not significantly smaller in the PBG when compared with the pulvinar while the amount of staining for synaptic markers was significantly different (Wilcoxon signed rank test, *p* < 0.05; Fig. 4*G*,*H*). Projections were not observed in other brain regions, thus confirming that Ntsr1+ cells in the SC exclusively target the pulvinar.

### Refining the molecular identity of Ntsr1+ neurons

Thus far, we have assumed that the Ntsr1-Tg mouse line unambiguously labels WFV, consistent with the prevailing practice in the field. However, newly identified markers for WFV enable a re-examination of this assumption. To critically examine the correspondence between the Ntsr1-Tg mouse line and recently identified molecular markers of WFV, we proceeded with fluorescent in situ hybridization of the following genes: *Necab1*, *Npnt*, and *Cbln2*. These candidates were selected based on their specificity in expression across depth and across molecularly defined clusters (Lein et al., 2007; Byun et al., 2016; Tsai et al., 2022; Liu et al., 2023). We performed three sets of experiments, one characterizing the expression of *Necab1*, *Cbln2,* and *tdTomato* in the Ntsr1-TgxAi14 mouse line; the second quantifying the overlap between *Cbln2*, *Necab1*, and *Npnt* in wild-type mice; and the last confirming the overlap of *Npnt* and *tdTomato* in the Ntsr1-TgxAi14 mouse line (Matcham et al., 2024). For all experiments, we examined the expression profile across the depth of the SC and the overlap between the molecular markers and reporter expression, *tdTomato*.

**Figure 5.**
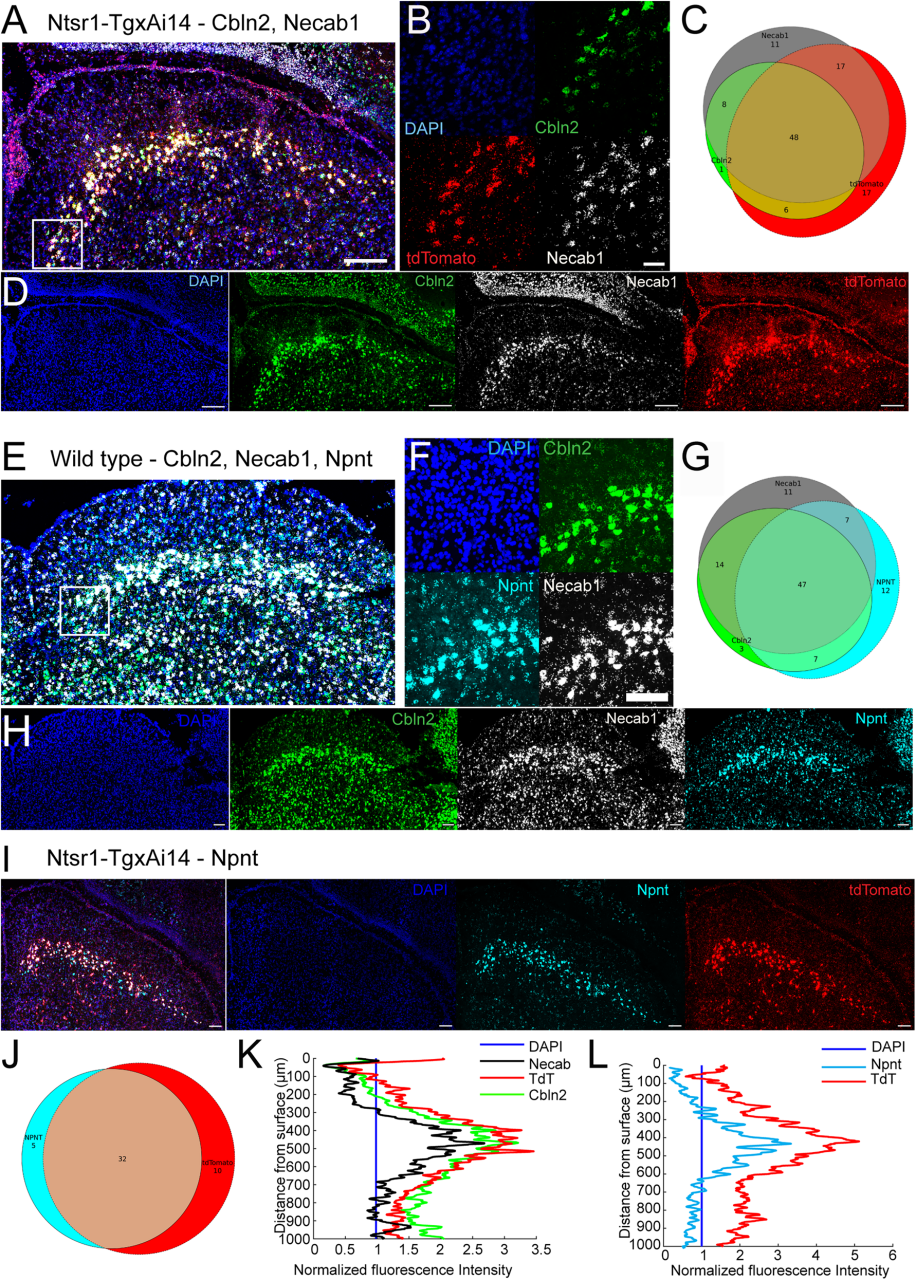
Ntsr1+ neurons expression of molecular markers. ***A***, In situ hybridization of *tdTomato*, *Cbln2*, and *Necab* in the Ntsr1-Tg mouse crossed with the reporter line Ai14 (Cre-dependent tdTomato). Overlay of the photomicrographs in ***D***, with the location of the high magnification presented in ***B*** highlighted. Scale bar, 100 µm. ***B***, High magnification of the stratum opticum of the SC from ***A***, where WFV somas can be found. Scale bar, 10 µm. ***C***, Venn diagram representing the average overlap in expression of the three probes at the single-cell levels. Three mice. ***D***, Photomicrographs of the SC, showing spatial organization in the expression pattern. ***E***–***H***, Same as ***A–D***. for in situ hybridization of *Npnt*, *Necab1*, and *Cbln2* in wild-type mice. ***I***, In situ hybridization of *Npnt* and *tdTomato* in the Ntsr1-Tg mouse crossed with the reporter line Ai14 (Cre-dependent tdTomato). Scale bar, 100 µm. ***J***, Venn diagram representing the average overlap in expression of the two probes at the single-cell levels. One mouse. ***K***, ***L***, Fluorescence intensity profile across the depth of the SC for (***K***) *tdTomato*, *Cbln2*, and *Necab1* and (***L***) *Npnt* and *tdTomato*.

First, we quantified the overlap between marker gene expression *Necab1*, *Cbln2*, and tdTomato reporter specifically in the SO for individual neurons in three animals for 1,184 cells (Fig. 5*A*–*D*). Partial overlap can be found in the expression of all the marker genes (Fig. 5*C*,*G*), with 66% of the cells expressing* tdTomato* expressing at least *Necab1* or *Cbln2* (787/1,184). Of all the*Necab1*expressing cells, 21% did not overlap with *tdTomato* expression (206/962), while 10% of *Cbln2* expressing cells did not overlap with tdTomato (102/962). Both tdTomato-negative populations intersected, with 22% expressing both *Cbln2* and *Necab1* (86/394). These results show that the majority of Ntsr1+ SC neurons intersect with at least **Necab*1* or *Cbln2*, while 45% (537/1,184) express both, which suggests subpopulations of WFV. To further explore the possibility that subpopulations of WFV can be defined based on molecular markers, we quantified the overlap of expression in wild types between *Npnt*, *Necab1*, and *Cbln2* (Fig. 5*E*–*H*). Forty-seven percent of cells located in the SO expressed all three markers. Interestingly, 11, 10, and 2% of cells expressed only *Npnt*, *Necab1*, and *Cbln2*, respectively, suggesting that *Cbln2* is the most commonly expressed marker among neurons located in the SO. We did not pursue extensive analysis of the overlap of *Npnt* expression and tdTomato driven by Ntsr1-Tg since these results have been previously reported (Matcham et al., 2024). Our results agree since we observed in one animal, an overlap of 76%, similar to the numbers reported by Matcham and colleagues. Our results confirm that the vast majority of Ntsr1+ SC neurons also express *Npnt*.

Depth profiles of molecular marker expression were obtained by averaging the fluorescence intensity from the dorsal surface to 1,000 µm in depth across the medial–lateral axis of sections. Expression was normalized against DAPI to account for differences in cellular density, and the lowest value encountered across depth was used for background subtraction. Using this method, we showed that all marker genes tested peaked in expression between 400 and 500 µm below the surface, and a decrease in fluorescence intensity was observed at the interface between the SC visual layers and deeper layer (Fig. 5*K*,*L*). Lower fluorescence intensity was observed in the SGS; however, for *Necab1* and *Cbln2*, *tdTomato* residual expression could be found below the SO, with twofold the intensity found in the SGS (0–200 µm below the surface against 800–1,000 µm). Of the different markers tested, *Npnt* has the most specific expression, with the lowest amount of expression in the deeper layers (50% increase in fluorescence of what is observed in the SGS). These results show that Ntsr1-driven *tdTomato* expression mostly overlaps with *Cbln2* and *Necab1*, while approximately a third of cells in the SO express either marker gene without expressing *tdTomato*. All these markers have peak expression in the SO, while *Npnt* shows the highest specificity with low expression in the deeper layers. *Npnt* also has the greatest overlap with* tdTomato* expression, while *Cbln2* and* Necab* might label WFV subpopulations that are not targeted by the Ntsr1-Tg mouse line. Taken together, these results confirm the higher specificity of *Npnt* for labeling WFV.

## Discussion

### Ntsr1+ SC neurons show specialized visual responses

This study shows that Ntsr1+ SC neurons respond to a variety of visual stimuli but on average prefer a small moving spot. These results mostly agree with previous studies that have investigated the response properties of WFV in vivo using electrophysiology in anesthetized preparation (Gale and Murphy, 2014, 2016) or fiber photometry (Brenner et al., 2023) and showed strong response to small slow-moving stimulus. Here, we were able to image a larger population, at single-cell resolution, within the same awake animal, display different types of visual stimuli, and analyze them for the same neuron. This allowed us to show that not all Ntsr1+ SC neurons show similar visual response preference, with some cells preferring for example looming rather than a small spot. Overall, neurons were visually responsive, with most neurons being responsive to at least one type of stimulus and showing an increase in responses during the presentation of a visual stimulus.

### Ntsr1+ SC neurons’ responses to looming are highly variable and modulated by locomotion

While characterizing the visual responses of Ntsr1+ SC neurons, a high variability in responses to looming was initially observed on a trial-to-trial basis. This variability is in sharp contrast with what we previously observed for direction-selective superficial neurons in the mouse SC (Savier et al., 2019) and, in the current study, what is observed for the same neurons in response to a moving spot. Here, we show that one factor that contributes to this variability is locomotion, which, surprisingly, only significantly affects visual responses to looming. It is worth noting that this effect varies across individuals, with some mice showing a higher modulation than others, which could not be associated with the sex of the animal. One of the limitations of our current study is that only locomotion was experimentally tracked, and the current data set does not include pupil size, which has been associated with arousal (for review, see Grujic et al., 2024). Indeed, arousal might contribute to the observed modulation due to the behavioral relevance of the looming stimulus, which mimics an approaching object (Yilmaz and Meister, 2013; De Franceschi et al., 2016). Another factor that could affect the responses is the “surprise” or saliency element of the visual stimulus, due to the high rate of change and increasing contrast, which is not found for other stimuli which display constant contrast across the duration of the presentation (Itti and Koch, 2000; Zhaoping, 2016; Veale et al., 2017; White and Munoz, 2017; Lee et al., 2020). To date, it remains unknown how this modulation by locomotion might affect brain-wide responses. WFV have been shown to contribute to cortical responses via the pulvinar, by either a surround suppression mechanism (Fang et al., 2008) or direct contribution (Beltramo and Scanziani, 2019; Brenner et al., 2023). Our previous study on the effect of locomotion in the SC did not show a strong modulation by locomotion for superficial direction-selective cells, while Schroeder and colleagues showed modulation of collicular neurons and retinal afferences (Schröder et al., 2020). Here, we argue that this modulation could be stimulus-, input-, and cell-type specific. Further investigations would allow us to isolate the afferences and cell types affected by locomotion, which could act in synergy with retinal inputs to modulate specific cell types and generate stimulus specificity.

### Sources of inputs to the Ntsr1+ SC neurons and modulation

Among retinal inputs, not all retinal ganglion cells innervate WFV. Reinhard and colleagues showed a preferential innervation by four types of RGC for SC neurons projecting to the pulvinar, which are presumably WFV, when compared with SC-PBG projecting neurons (Reinhard et al., 2019), while Tsai and colleagues showed that mostly alphaRGCs target WFV (Tsai et al., 2022). Another likely candidate for the modulation is the PBG, a cholinergic nucleus which has been associated with modulation of the SC (Deichler et al., 2020; Basso et al., 2021; Peng et al., 2024). Furthermore, cortical inputs from the primary visual cortex to the SC have been shown to enhance visual responses to looming in awake animals, making them also a likely candidate for the reported modulation (Zhao et al., 2014). Other studies have also shown that cortical inputs preferentially target WFV in the SC and that these projections are excitatory (Masterson et al., 2019; Jiang et al., 2023). Among the other inputs to Ntsr1+ SC neurons, the vLGN has been implicated in the modulation of responses to looming via projection to the SC and behavioral modulation (Fratzl et al., 2021; Salay and Huberman, 2021, Vega-Zuniga et al., 2025), thus placing it as another likely candidate for modulation by locomotion.

### Preferences for upward direction

Multiple studies have reported direction preference bias in the SC, with different types of patterns observed, from concentric organization to clusters of preferred orientation. The discrepancy observed in the organization has been in part explained by the anesthesia state of the animal, the stimulus type used, and location but could also be explained by the depth at which the recordings were performed and the cell types from which the results were acquired (Ahmadlou and Heimel, 2015; Feinberg and Meister, 2015; de Malmazet et al., 2018; Kasai and Isa, 2022). Here, using the same methods as previously used where we could not observe stereotypy in direction preference distribution (Savier et al., 2019), we observed a bias toward upward direction when focusing exclusively on Ntsr1+ SC neurons. This observation is similar to what was originally observed by Dräger and Hubel (1975) using electrophysiological studies and Gale and Murphy's original investigation on Ntsr1+ SC neurons (Gale and Murphy, 2014). Here, we argue that direction-selective cells observed at the dorsal surface of the SC encode all cardinal directions, while other cell types, which are located deeper, could show bias in their preferred direction as a function of location. One of the limitations of the current study is the surgical preparation used, which limits access to the caudal–medial part of the SC and does not allow the investigation of response properties of Ntsr1+ SC neurons in other parts of the visual field.

### Ntsr1+ SC neurons molecular heterogeneity

Investigation in other species has shown that at least two types of WFV could be found in mammals (Major et al., 2000; Fredes et al., 2012; Zhou et al., 2017). These types can be distinguished by their projection pattern, either ipsilateral or bilateral, and morphology. Since most studies in mice investigating the WFV have been conducted using the Ntsr1-Tg mouse line, one cannot exclude that this mouse line only labels a subpopulation of WFV. Our current results show that a proportion of cells localized in the SO layers express either *Cbln2* or *Necab1* and are not labeled by the Ntsr1-Tg mouse line. Further investigation is required to determine if these *Cbln2*+/*Necab1*+/Ntsr1− neurons are indeed WFV and have distinct morphologies and projection patterns. The substantial overlap between *Npnt*- and Ntsr1-Tg-driven tdTomato expression suggests that both the Ntsr1-Tg mouse line and* Npnt* label the same population of WFV. We also confirmed the higher specificity of *Npnt* expression pattern to the WFV and would suggest further studies investigating the role of the WFV, especially in behavior, to use the Npnt-FlpO mouse generated by Duan's group (RRID:IMSR_JAX:034305; Tsai et al., 2022; Matcham et al., 2024). This mouse line would avoid potential confounds when using viral injections which could also transfect the deeper layers, the inferior colliculus, or by retrograde transport, the retinal ganglion cells. Indeed, *Npnt* is expressed very sparsely in the GCL compared with Cre in the Ntsr1-Tg (Su et al., 2021).

Overall, our result showed an unexpected stimulus-dependent modulation by locomotion and interindividual variability for a population of well-defined neurons in the SC, the WFV. Further studies would reveal what aspects of looming are required to evoke this modulation and delineate the contribution of internal state to this modulation. The identification of the projections driving this modulation would also be of prime interest, furthering our understanding of how internal state and ethological relevance affect our perception of sensory information.
